# Cerebral Embolism due to a Large Papillary Fibroelastoma arising from the Coumadin Ridge

**DOI:** 10.1002/ccr3.2178

**Published:** 2019-05-10

**Authors:** Teppei Kamimura, Kanta Tanaka, Hiroshi Yamagami, Masatoshi Koga

**Affiliations:** ^1^ Department of Neurology National Cerebral and Cardiovascular Center Osaka Japan; ^2^ Division of Stroke Care Unit National Cerebral and Cardiovascular Center Osaka Japan; ^3^ Department of Cerebrovascular Medicine National Cerebral and Cardiovascular Center Osaka Japan

**Keywords:** cerebral embolism, papillary fibroelastoma, cardiac tumor, coumadin ridge

## Abstract

A 70‐year‐old woman developed acute cerebral infarction. Transthoracic echocardiography showed a large mobile mass in the left atrium, suggesting cardiac myxoma as the most likely diagnosis. Surgical exploration revealed a papillary fibroelastoma originating from the coumadin ridge, which is fairly rare but important as a source of cerebral embolization.

## 1 CASE SUMMARY

**Figure 1:**
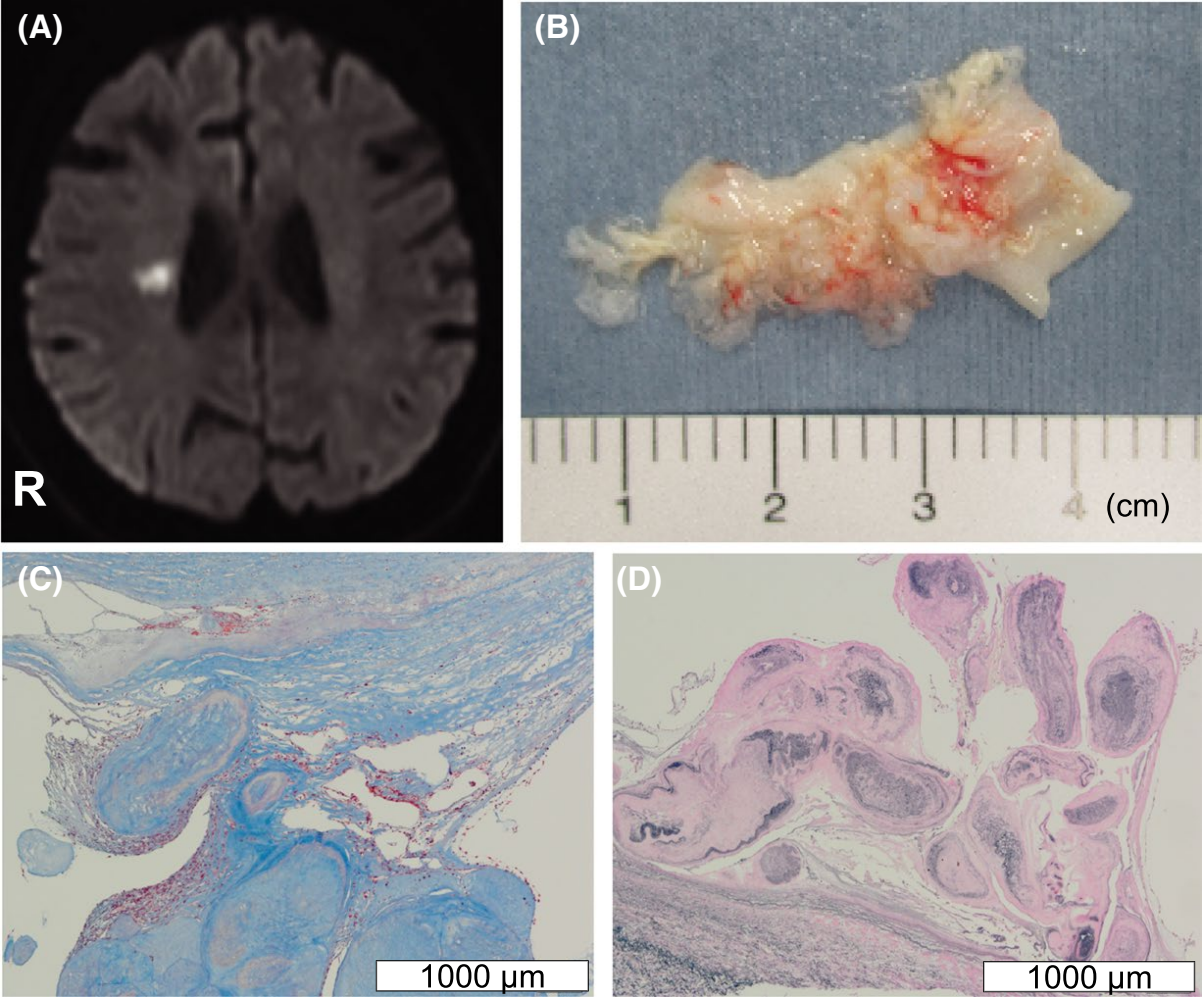
Diffusion‐weighted imaging. Acute infarction in the right corona radiata is shown (A). Gross inspection of the resected tumor with a whitish, elastic, and papillary appearance (B). Masson trichrome staining showing papillary collagenous cores (C). Elastica van Gieson staining showing the layer of elastic fibers covered with endothelial cells (D)

A 70‐year‐old afebrile woman with no history of malignancy suddenly developed paresis of the left lower limb. Magnetic resonance imaging showed a small acute infarction in the right corona radiata. Urgent bedside transthoracic echocardiography showed a highly mobile, fluffy mass in the left atrium (Video S1, Figure 1). The large mass size suggested cardiac myxoma as the most likely diagnosis.^1^ Immediate surgical exploration to prevent further embolization showed the mass originating from the coumadin ridge, with histological confirmation of papillary fibroelastoma (Figure 1). Papillary fibroelastoma arising from the coumadin ridge is fairly rare but important as a source of cerebral embolization.^2^

## CONFLICT OF INTEREST

None declared.

## AUTHOR CONTRIBUTION

Teppei Kamimura and Kanta Tanaka: acquired and interpreted the data, drafted the manuscript. Hiroshi Yamagami and Masatoshi Koga: acquired and interpreted the data, critically revised the manuscript for intellectual content.
